# Ranking’s Half-Year Abstract of the Medical Sciences

**Published:** 1850-11

**Authors:** 


*Ranking's Half-Year Abstract of the Medical Sciences. No.* 11. *January to July*, 1850.

*Braithwaite's Retrospect of Practical Medicine and Surgery. Part the Twenty-first.* 1850.

These popular periodicals continue to be issued punctually, and are promptly reproduced in this country. They are too well known to require aught from the journalist save the announcement of the appearance of the successive numbers.

The work by Braithwaite is issued by Daniel Adee, 107 Fulton-st., New York. Ranking's Abstract is republished by Lindsay & Blakiston, Philadelphia.

